# Late-Developing Supernumerary Premolars: Analysis of Different Therapeutic Approaches

**DOI:** 10.1155/2016/2020489

**Published:** 2016-09-28

**Authors:** Sergio Paduano, Roberto Rongo, Alessandra Lucchese, Domenico Aiello, Ambrosina Michelotti, Cristina Grippaudo

**Affiliations:** ^1^Department of Health, “Magna Graecia” University of Catanzaro, Viale Europa, Loc. Germaneto, 88100 Catanzaro, Italy; ^2^School of Orthodontics, Department of Neurosciences, Reproductive Sciences and Oral Sciences University of Naples “Federico II”, Via Pansini 5, 80131 Naples, Italy; ^3^Department of Orthodontics, University Vita Salute San Raffaele, Milan, Italy; ^4^Research in Dentofacial Orthopedics, Orthodontics and Pediatric Dentistry, Research Area in Oral Pathology and Implantology, IRCCS San Raffaele Hospital, Via Olgettina 58, 20132 Milan, Italy; ^5^Postgraduate School of Orthodontics, Università Cattolica del Sacro Cuore, Largo A. Gemelli 8, 00168 Rome, Italy

## Abstract

This case series describes the different potential approaches to late-developing supernumerary premolars (LDSP). LDSP are supernumerary teeth (ST) formed after the eruption of the permanent dentition; usually they develop in the premolar region of the upper and lower jaw. The choice to extract or to monitor the LDSP depends on many factors and has to be carefully planned due to the several risks that either the monitoring or the extraction could provoke. These four cases of LDSP showed different treatment plan alternatives derived from a scrupulous assessment of the clinical and radiographic information.

## 1. Introduction

Supernumerary teeth (ST) or hyperodontia is one of the less frequent developmental anomalies characterized by an excess number of teeth with respect to the usual configuration of 20 deciduous and 32 permanent. The prevalence of ST varies between 0.1% and 3.8% [1]; they are more reported in the permanent dentition (1–3% of general population) than in primary dentition (0.8% of population) [2]. The etiology of ST is still not completely understood. Several theories were proposed to explain this condition such as the phylogenetic theory [3], the dichotomy theory (splitting of the tooth germ) [4], and the hyperactivity theory (hyperactive dental lamina) [5]; however, it is most likely to be due to a combination of genetic and environmental effects [6]. In fact, due to the higher prevalence of ST in male than female (2 : 1 ratio) an X linked hereditary pattern was presumed [7]. Moreover, certain genetic related syndrome, cleidocranial dysplasia, Gardner's syndrome [8, 9], and cleft lip and palate [10] predispose to ST.

Chronology, topography, and morphology are used to classify hyperodontia. Based on chronology, ST can develop before the primary dentition (*predeciduous*), contemporary to the permanent teeth, or after the permanent dentition (*post-permanent dentition*). Frequently patients are affected by a single supernumerary (76–86%), less by double supernumeraries (12–23%), and rarely by multiple supernumeraries (<1%) [7]. ST are more frequent in premaxilla region but can occur also in the mandible, where the most common ST recurred in the premolars region. Supernumerary premolars represent between 8 and 9.1% of all supernumerary teeth [11] and often look like normal premolars and develop after the formation of the permanent teeth [12].

ST may cause some complications including delayed eruption and/or displacement of permanent teeth, root resorption, and cyst formation [13, 14]. Moreover, the stability of the results is an important objective of the orthodontic treatment [15]; specifically, post-permanent ST can also compromise this stability and interfere with orthodontic closure mechanics or with implant and mini-implant placement [1]. Generally, supernumerary teeth are detected incidentally during radiographic examination, and late-developing supernumerary premolars (LDSP) are often detected at the end of an orthodontic treatment, because the age of formation is around 12-13 years. There are two options in case of LDSP, follow-up or extraction; and there are several factors to analyze and choose the best therapeutic option. Modern radiographic technologies as three-dimensional computed tomography (3D CT) and cone beam computed tomography (CBCT) allow a better assessment of the risk to extract or not to extract a supernumerary post-permanent tooth and a more detailed analysis of the stomatognathic system's bone structure [16, 17].

There are some reports on LDSP in the literature [12, 18–23], and four further cases of LDSP with different approaches for their management are described in this paper, to improve knowledge regarding how to choose the best therapeutic option.

## 2. Case 1

### 2.1. Diagnosis and Etiology

**Figure 1:**
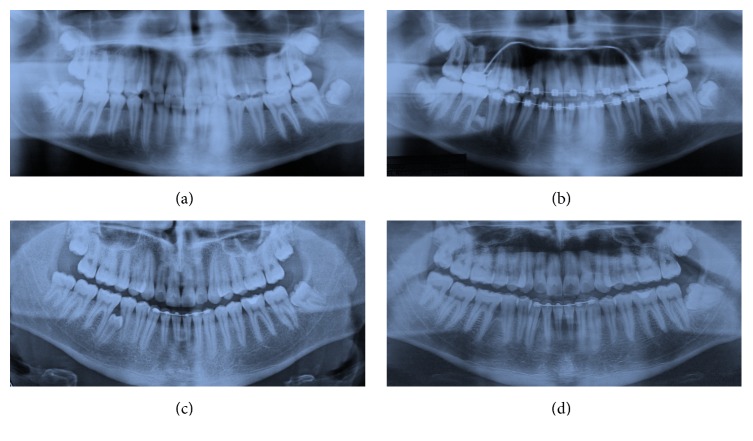
Case 1 radiography: before treatment 2004 (a), before rebonding, ST in 4.5–4.6 region, 2006 (b), 5 years after debonding 2011 (c), and after the extraction of the ST 2013 (d).

A 12-year-old male in the permanent dentition was brought to our clinic for orthodontic treatment. The patient presented Class II, Division 2 malocclusion on Class I skeletal base. The overjet was reduced (1 mm) and the overbite increased (5 mm). Medical and familiar histories were unremarkable. The pretreatment radiography showed the presence of all the permanent teeth with also the presence of the wisdom teeth (Figure 1(a)). Treatment started in 2004 with thermoelastic wires to have a less painful resolution of the crowding [24]. During the treatment, after two years from the start, a new panoramic radiograph was requested to evaluate the necessity of some brackets rebonding, and ST between 4.5 and 4.6 was detected. The radiography showed a bicuspid crown with a dental follicle (Figure 1(b)).

### 2.2. Treatment Objectives

The treatment objective was to avoid the damage of the surrounded tissues and of the contiguous teeth and the disruption of the occlusion, balancing the risk/benefit ratio between the follow-up of the tooth and the extraction.

### 2.3. Treatment Alternatives

There are two options when a LDSP is present; the first one is in the radiographic follow-up and the second one is the extraction. In this case the OPG (Figure 1(b)) revealed that the tooth was still developing, only the crown being formed, and that the tooth did not have any contact with the contiguous teeth. Performing an extraction of this tooth implied a germectomy with a deep mandibular access and a great bone loss. Moreover, no further active orthodontic treatment has to be performed in the lower arch. Hence, due to these considerations the best choice was to monitor the tooth during the time.

### 2.4. Treatment Progress

The patient's parents were informed about the LDSP. Clinical and radiographic follow-up were performed to verify the onset of any complication. After 5 years the tooth was still not completely formed but an initial repositioning of the root of 4.5 was present (Figure 1(c)). In 2013, when the tooth achieved a more accessible position it was extracted upon the request of the patient (Figure 1(d)).

## 3. Case 2

### 3.1. Diagnosis and Etiology

**Figure 2:**
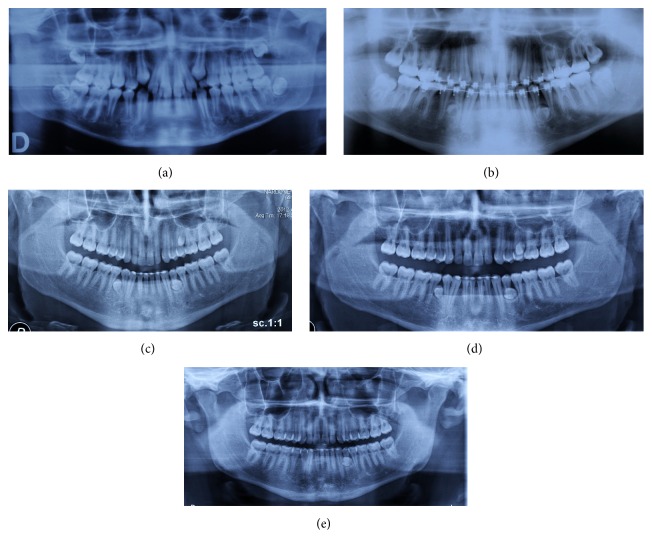
Case 2 radiography: before treatment 2006 (a), before rebonding, ST in regions 3.4–3.5, 4.4–4.5, and 2.5–2.6, 2009 (b), 3 years after debonding 2012 (c), 4 years after debonding 2013 (d), and after the extractions of ST in regions 4.4–4.5 and 2.5–2.6, 2015 (e).

An 11-year-old male in the mixed dentition was referred to the clinic for an orthodontic treatment. He presented Class II, Division 1 malocclusion on Class II skeletal base. The overjet and overbite were increased, and a molar crossbite was present [25]. Medical and familiar histories were unremarkable; no one of the parents had ST. The pretreatment radiography showed the presence of all the permanent teeth with also the presence of the wisdom teeth and the upper canine in eruption phase (Figure 2(a)). The patient was treated by means of aesthetic brackets and wires [26]. In the final stage of the orthodontic treatment in the panoramic radiograph, requested to assess the root parallelism, three supernumerary premolars, two in the lower jaw and one in the upper jaw, were identified (Figure 2(b)).

### 3.2. Treatment Objectives

The treatment objectives are to avoid that the ST could damage the surrounded tissues and the contiguous teeth and disrupt the occlusion, balancing the risk/benefit ratio between the follow-up of the tooth and the extraction.

### 3.3. Treatment Alternatives

In this case, the first treatment approach was the follow-up. Even if the ST in regions 3.4–3.5 and 4.4–4.5 created a root displacement with possible resorption, in the upper jaw ST in 2.5–2.6 region did not create any issue. However, due to the very close relationship between the lower jaw supernumerary and the mental foramen it was preferred also in this case to monitor the teeth over time.

### 3.4. Treatment Progress

The patient's parents were informed about the LDSP. The patient was subjected to follow-up, but his participation was not very regular, and in 2012 the OPG showed the formation of the three teeth (Figure 2(c)); in 2013 two out of the three teeth erupted and due to the more accessible position it was decided to extract these two teeth (Figure 2(d)). In 2015 the patient has still the supernumerary in 3.4–3.5 region (Figure 2(e)).

## 4. Case 3

### 4.1. Diagnosis and Etiology

**Figure 3:**
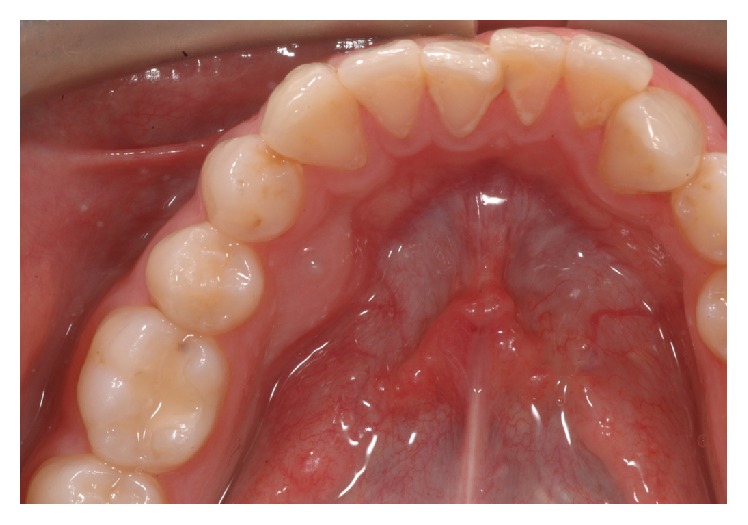
Case 3 intraoral image: lingual bulge in 4.4–4.5 region.

**Figure 4:**
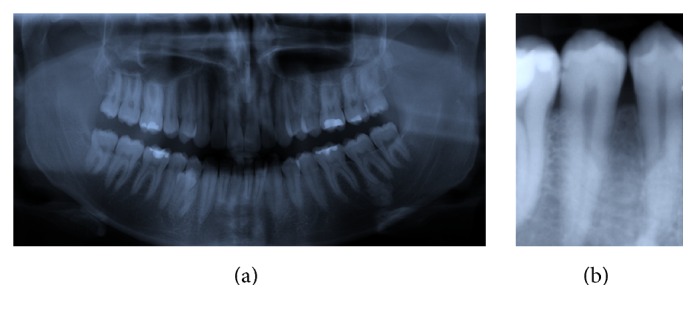
Case 3 radiography: ST in 4.4–4.5 region 2015 (a) and after extraction 2015 (b).

A 25-year-old male patient in permanent dentition presented to the orthodontic clinic with Class II subdivision on Class I skeletal base, with crooked teeth. At the clinical examination, the patient presented a little bulge in the 4.4–4.5 region (Figure 3) and at the panoramic radiography the presence of a supplemental tooth in this region was discovered (Figure 4(a)). The tooth was completely formed; the root apex was closed and seemed not to create any damage to the circumstance region.

### 4.2. Treatment Objectives

The treatment objective is to start an orthodontic treatment in the upper and lower jaw.

### 4.3. Treatment Alternatives

In this case, the only possible choice was the extraction of the ST. The patient came to the clinic to correct his malocclusion by means of an orthodontic treatment. Hence, due to the will of the patient to start an orthodontic treatment, the extraction was the best choice.

### 4.4. Treatment Progress

**Figure 5:**
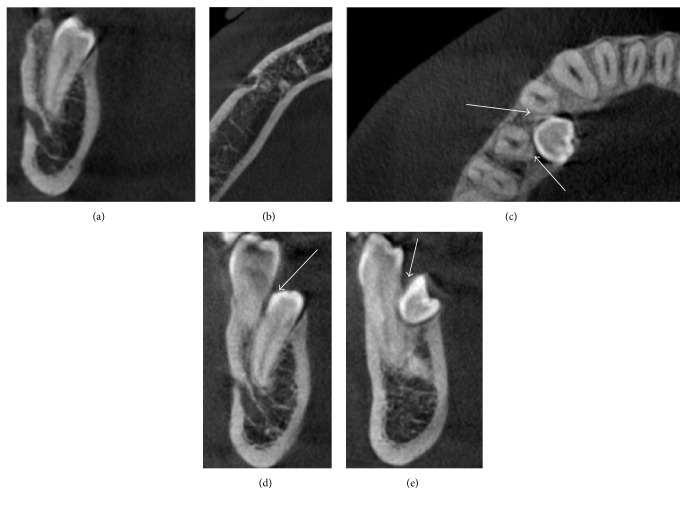
Case 3 CBCT: no continuity between the ST's root and the alveolar nerve (a, b) and root resorption of contiguous teeth, indicated by the white arrows (c–e).

Before extracting the tooth, it was decided to perform a CBCT of the lower arch to assess the relationship between the ST and the mandibular nerve. Examining the CBCT ascertained that there was no contiguity between the tooth and the nerve (Figures 5(a) and 5(b)) and that the roots of 4.4 and 4.5 were damaged (Figures 5(c)–5(e)). Hence, due to the favorable tooth position, the root damage, and the patient's will, the extraction was performed. After the extraction a periapical radiograph of the extraction site was done (Figure 4(b)); then the patient started the orthodontic treatment.

## 5. Case 4

### 5.1. Diagnosis and Etiology

**Figure 6:**
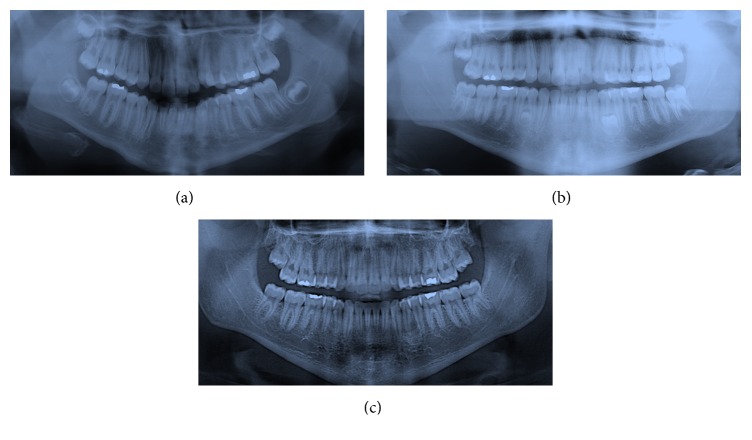
Case 4 radiography: before treatment 2011 (a), after treatment, ST in regions 3.4–3.5 and 4.4–4.5, 2014 (b), and after extraction of ST 2014 (c).

**Figure 7:**
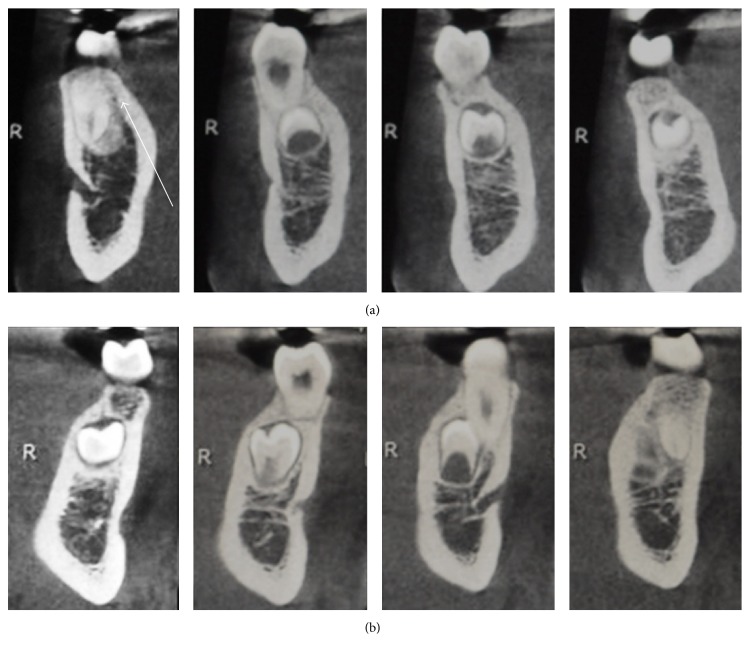
Case 4 CBCT: root resorption of contiguous teeth, indicated by the white arrows ((a) 4.3–4.4 and (b) 3.4–3.5).

 A 14-year-old patient, in good health, visited the clinic in March 2011. The diagnosis was dentoskeletal Class II malocclusion, with greatly increased overjet, atypical swallowing with tongue thrust, and anterior open bite. Initial OPG revealed all the permanent elements including gems of wisdom teeth with the absence of any number or shape of dental anomalies (Figure 6(a)). The patient was treated with self-ligating brackets [27] for 36 months. In the final OPG in March 2014, two supernumerary teeth in 3.4–3.5 and 4.4–4.5 area were found (Figure 6(b)). A CBCT of the lower arch was performed to better understand the ST position, the relationship with the surrounded tissues, and the presence of any root resorption that were not visible in the OPG. From the CBCT, the root displacement of 3.4 and 4.4 and the root resorption of 4.4 were clear (Figures 7(a) and 7(b)).

### 5.2. Treatment Objectives

The treatment objectives are to avoid further damage of the contiguous teeth and of the surrounded tissues and to maintain a good occlusion, balancing the risk/benefit ratio between the follow-up of the tooth and the extraction.

### 5.3. Treatment Alternatives

Once root displacement and root resorption were found on the CBCT no other choice than extraction was possible. In this case, even if the ST are in a deep position and the access will remove healthy bone, the damage present on the roots suggests extracting the ST to prevent worsening of the root resorption.

### 5.4. Treatment Progress

The patient's parents were informed about the LDSP. The surgical intervention was planned with the help of the CBCT to preserve the bone. For the ST in 3.4–3.5 region the surgical access was on the lingual side of the lower jaw; instead, for the ST in 4.4–4.5 region the access was buccal, paying attention to the mental foramen.

The OPG after the extraction (Figure 6(c)), again, did not show any root resorption that was possible to identify only with the CBCT due to the slight damage and the close superimposition between the ST and the teeth.

## 6. Results

In all the four cases presented the treatment objectives were achieved. In the first two cases where a follow-up approach was chosen, the occlusion remains stable over time. However, thanks to the radiographic images it was possible to check the evolution of the ST and to decide, in case of any complication, to extract the tooth as in Cases 1 and 2. In Cases 3 and 4 the aims were different; in Case 3 the extraction was mandatory both for the patient's will to start an orthodontic treatment and for the little bulge present in the lower jaw. Also in Case 4 the extraction was mandatory because contiguous teeth showed root resorption, even if more dangerous than in the other cases; however the extractions stopped this phenomenon and the treatment aim was reached.

## 7. Discussion

Several cases of LDSP are discussed in the literature [12, 18–23]; hence, even if uncommon, this condition has to be taken into account in the different phases of an orthodontic treatment. It was reported that the physiological calcification of the premolars starts between the age of 1.5 and 2.5 [28], although usually they are not detectable in radiograph until 3 or 4 years [29]. Otherwise, as reported in the literature, supernumerary premolar teeth start their developing around 10–15 years, a critical age since many children have an orthodontic treatment at that time [12, 18–23]. In fact, in all 4 cases reported, the supernumerary teeth were not detected in patients younger than 14 years old.

There are two solutions after discovering a LDSP, extracting or monitoring. The advantages and disadvantages of both options are different and the final decision has to be taken considering the risk/benefit relationship of the surgical removal. As a matter of fact, a ST could delay the eruption of the adjacent teeth, alter the eruption direction, displace the adjacent tooth, or cause cystic lesion and root resorption [30, 31]. Nevertheless, also the surgical removal is not totally free of risks; extraction of impacted teeth may lead to damage to adjacent structures and/or adjacent teeth with possible ankyloses [30, 31]. Hence, only in the case that the benefits of removal overbalance the risks of surgery, the teeth have to be extracted.

Bodin et al. [32] reported that only 2 percent of supernumeraries in the premolar region exhibited any pathological change and suggested that the teeth may be left rather than the risk of surgical damage.

The choice to extract or not to extract the supernumerary teeth was due mainly to the damage that the tooth left in locus might cause. In Case 1, the decision was to monitor the lower jaw supernumerary tooth because it was in a position that unlikely evolves in a problematic situation and did not disrupt the occlusion. The tooth was extracted only due to the patient's request and for the slight repositioning of the root of the 4.4. Instead, in Case 2 the solution to monitor the development of the teeth was due to the dangerous position of the teeth close to the mental foramen, thus being difficult to extract. In this case, once the teeth were totally formed and were more accessible the teeth were removed. In the third case, the tooth was extracted before starting the orthodontic treatment and the CBCT helped the clinician to plan the surgical approach and to diagnose the root resorption due to the LDSP. The last case was an example of how important could the 3D imaging be to better evaluate the presence of damage of the adjacent structures due to ST. Probably also in Case 1 a CBCT could have shown root resorption in 4.5 or 4.6.

Therefore, the correct management of a supernumerary tooth depends on risk/benefit relationship between surgical extraction and monitoring, the type and the position of the tooth and the possible damage in the surrounded tissues, and the orthodontic treatment stage in which the tooth was discovered. In many cases only 3D imaging could help the clinician to clearly evaluate the problem.

## 8. Conclusions

The cases highlight the late development of supernumerary teeth in the premolar region at different times with respect to the orthodontic treatment. If the post-permanent tooth appears before starting the orthodontic treatment, the suggestion is to remove it to avoid interferences during the teeth movement. The same protocol should be used even when the tooth was discovered during active orthodontic treatment. On the other hand, if the tooth was detected at the end of the orthodontic treatment or with no need of orthodontic treatment and did not disrupt the patient occlusion, it should be monitored by means of a regular radiographic and clinical follow-up, and the extraction has to be an option only if the risk/benefit relationship is favorable.
